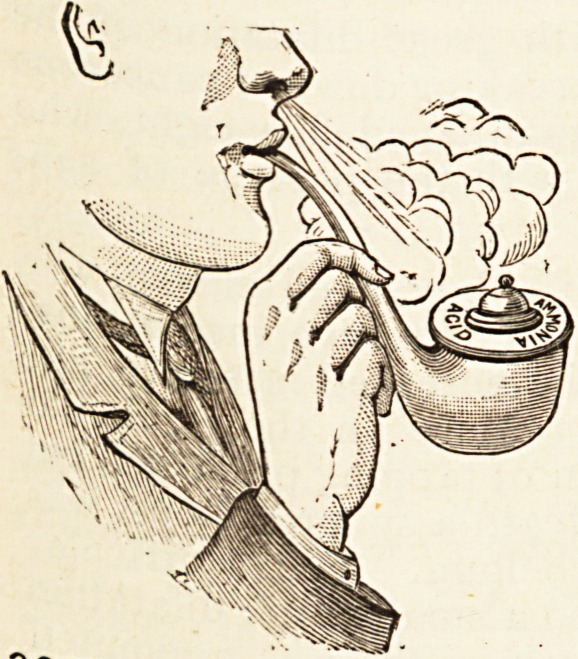# Notes on Preparations for the Sick

**Published:** 1893-03

**Authors:** 


### Notes on Preparations for the Sick.

---

**Foods for Infants.**—ALLEN & HANBURYS, London.—Three foods for infants are prepared by this firm. The first, which is called *First Food for Infants*, is intended to be used till the end of the third month. During the next three months the second, called *Mother's Milk Food*, may be used. The third, described as *Malted Food*, is specially designed for children of six months of age and upwards. The first two are complete in themselves, and require only the addition of hot water. They are also sterilized, and are uniform in composition. The second differs from the first in having some additional maltose and a small proportion of dextrine, with some soluble phosphates, resulting from the mashing of crushed whole meal with barley malt. They are concentrated *in vacuo*, and packed in close vessels. No unconverted starch exists in them. The Malted Food has for its basis fine wheaten flour, which has been subjected to heat and to the action of a proportion of malt extract sufficient to ensure the conversion of a large proportion of the starch.

---

**Peptonising Tablets.**—FERRIS & Co., Bristol.—These tablets contain 2½ grains of pancreatin, with 8 grains of sodium bicarbonate. Peptonisation takes place rapidly at a temperature of about 105°, and the following instructions for use cannot be repeated too frequently, as so many mistakes are commonly made in the execution of so simple a chemical process: A quarter of a pint of water is added to a pint of milk, and the whole divided into two equal portions, of which one is raised to the boiling point and the other is reserved. The reserved cold half is then mixed with the boiling half, and one of the peptonising tablets, reduced to powder by crushing it with a spoon, is added, and the whole stirred briskly. The jug containing the milk is then placed under a "cosey" to retain the heat, and left for about an hour to an hour and a half, when the milk is finally boiled for two or three minutes to prevent the development of the bitter taste. The preparation is rendered more palatable if the cream is skimmed off first and put aside, and when the milk has been peptonised, added to it.

---

**Bi-palatinoids of Ferrous Carbonate (Blaud's Pill).**—OPPENHEIMER, SON & Co., London.—If Blaud's pill be the thing, surely this preparation must be the prince of all. In these palatinoids, the incompatible chemicals are separated from each other by a thin septum of soluble jujube; in the stomach, the desired salt is formed perfectly fresh and unoxidised. Wherever the ferrous carbonate in moderate dose is indicated these bi-palatinoids will supply it, and with a minimum of the draw-backs usually associated with iron.

---

**Syrup Iron Chloride.**—PARKE, DAVIS & CO., Detroit.—Each fluid ounce of this preparation contains 40 drops of the United States tincture of chloride of iron. The presence of free acid is unnecessary for perfect solution of the basic salts of iron, and the therapeutic efficacy of the tincture is not diminished by the removal of the excess of acid, which is commonly believed to have a destructive action on the teeth.

---

**Ammonium Chloride Inhaler (Dr. Stanson Hooker's).** — BURROUGHS, WELLCOME & CO., London.—Ammonium chloride inhalers have not yet outlived their reputation. We have often advised the wife to produce her inhaler when the husband lights up his pipe; but this new "pipe" form of the apparatus will enable the wife to blow a cloud which will surpass that of her spouse. The accompanying picture sufficiently explains the instrument.

---

**Urethral Antrophors.**—THOMAS CHRISTY & CO., London.—Under the above title we have received some specimens of coated spring bougies, charged with various medicaments for the treatment of gonorrhœa, and so constructed as to permit their introduction into the urethra with ease and without discomfort. They are coated with various strengths of iodoform, thallin, sulphate of zinc, &c., so as to render them especially useful in the more chronic forms of urethritis, which affect chiefly the deeper parts of the urethra. They are beautifully made, and are approved by all who have tried them.

## Figures and Tables

**Figure f1:**